# Imaging findings of intra-articular tumor/tumor-like lesions based on pathologic correlation

**DOI:** 10.1007/s11604-025-01928-w

**Published:** 2025-12-19

**Authors:** Jun Tsukamoto, Akitaka Fujisaki, Koichiro Futatsuya, Yuki Koreeda, Kazuhiro Kajio, Sayaka Inoue, Yoshiko Hayashida, Akinori Sakai, Masanori Hisaoka, Yoshinao Oda, Takatoshi Aoki

**Affiliations:** 1 Department of Radiology, School of Medicine, University of Occupational and Environmental Health, 1-1 Iseigaoka, Yahatanishi-ku, Kitakyushu, 807-8555 Japan; 2 Department of Orthopaedic Surgery, School of Medicine, University of Occupational and Environmental Health, 1-1 Iseigaoka, Yahatanishi-ku, Kitakyushu, 807-8555 Japan; 3 Department of Pathology and Oncology, School of Medicine, University of Occupational and Environmental Health, 1-1 Iseigaoka, Yahatanishi-ku, Kitakyushu, 807-8555 Japan; 4 Department of Anatomic Pathology, Graduate School of Medical Sciences, Kyushu University, 3-1-1, Maidashi, Higashi-ku, Fukuoka-shi, Fukuoka-ken, 812-8582 Japan

**Keywords:** Intra-articular tumor, Intra-articular tumor-like lesion, Radiograph, Magnetic resonance imaging (MRI), Synovium

## Abstract

Proper treatment of intra-articular tumor/tumor-like lesions (tenosynovial giant cell tumor, synovial chondromatosis, synovial hemangioma / intra-articular venous malformations, lipoma arborescens, etc.) depends on an accurate diagnosis. This review highlights the imaging findings of intra-articular tumor/tumor-like lesions and the other synovial diseases (gout, amyloid arthropathy, rheumatoid arthritis, ganglion, and postoperative intra-articular tumor) to determine whether they could help in establishing the correct diagnosis. Many synovial proliferative diseases have specific imaging characteristics and an awareness of these characteristics along with their pathological and anatomical features can allow for an accurate diagnosis. Even though a wide spectrum of diseases may involve the synovium, careful MRI assessment used in conjunction with clinical information can lead to a substantial narrowing of the differential diagnosis.

## Introduction

The differential diagnosis of intra-articular masses is diverse, and accurate characterization is essential for guiding appropriate management. Intra-articular tumors and tumor-like lesions, such as tenosynovial giant cell tumor, synovial chondromatosis, synovial hemangioma / intra-articular venous malformations, lipoma arborescens, exhibit distinct clinical and imaging features that reflect their underlying pathology. Timely and accurate identification of these lesions can significantly influence treatment decisions and outcomes. A thorough understanding of synovial and joint anatomy is critical for radiologists in determining the origin of intra-articular soft tissue masses, especially in anatomically complex joints. This anatomical knowledge aids in narrowing the differential diagnosis by correlating lesion location with typical disease patterns. Furthermore, intra-articular tumors and tumor-like conditions often demonstrate specific imaging characteristics on MRI and other imaging modalities. However, these lesions may sometimes mimic non-neoplastic synovial pathologies including bursitis, deposition diseases (e.g., gout, amyloid arthropathy), autoimmune conditions (e.g., rheumatoid arthritis), and miscellaneous entities such as ganglion or cyclops lesions. Metastatic disease or primary sarcoma involving the joint space is rare but need to be considered in the differential diagnoses.

The purpose of this review is to present a comprehensive overview of the imaging features of intra-articular tumors and tumor-like lesions, alongside a discussion of other synovial pathologies that may present with similar imaging appearances. Emphasis is placed on radiologic-pathologic correlation and anatomical localization, with the goal of improving diagnostic confidence and accuracy in clinical practice.

## Anatomy

**Fig. 1:**
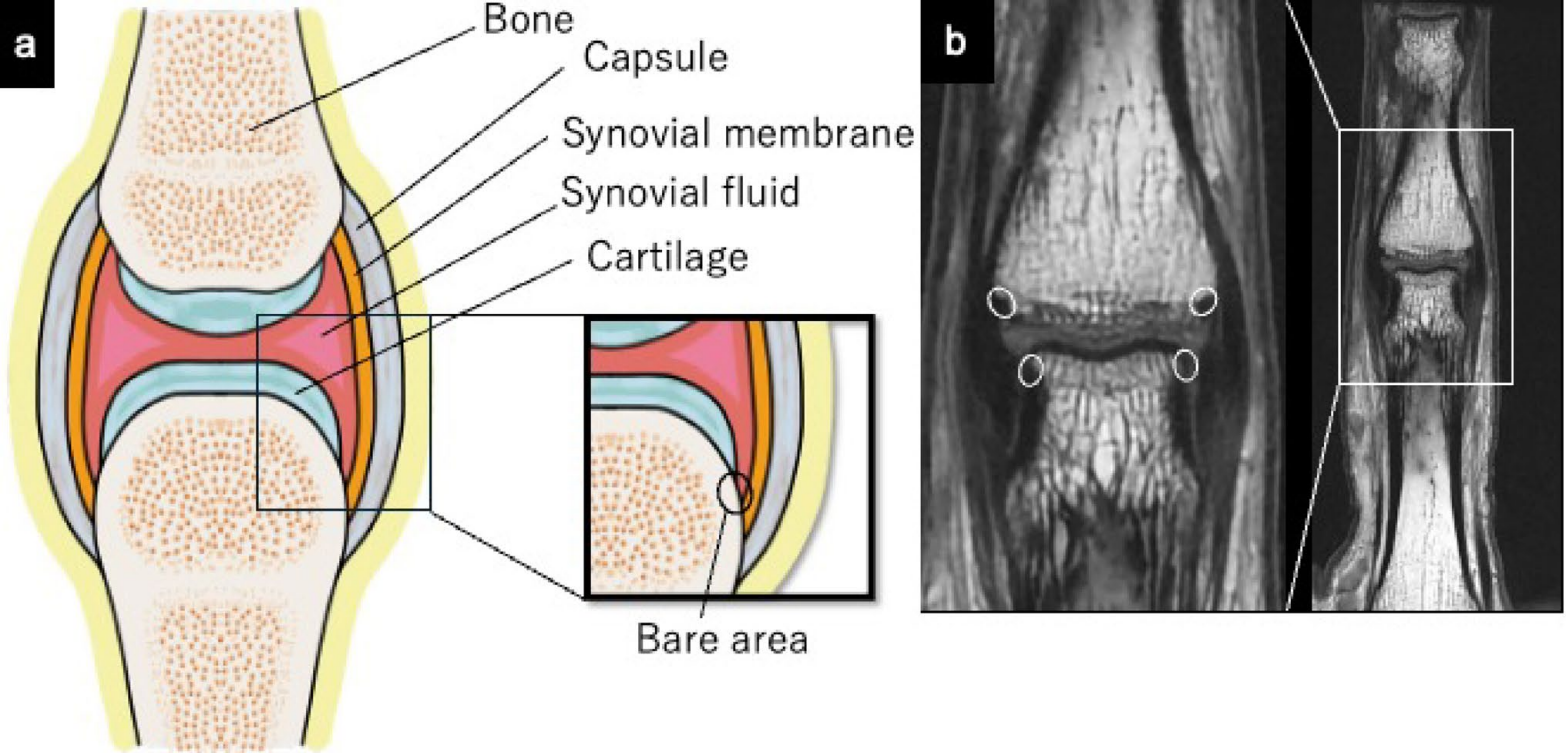
In a synovial joint, the surface of the articulating bones is normally covered with hyaline cartilage, but a small region called the “bare area” exists between the insertion of the joint capsule and the edge of the cartilage (circle area), where the bone is directly covered by synovium

**Fig. 2:**
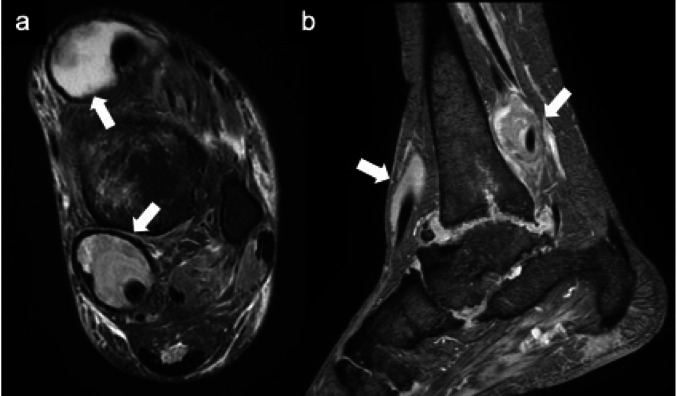
Rheumatoid arthritis Axial fat-suppressed T2-weighted MR image (**a**) and sagittal T2*-weighted MR image (**b**) show distention of the tendon sheaths (arrows). The high signal intensity surrounding the tendons indicates fluid accumulation within the tendon sheaths, suggesting tenosynovitis

**Fig. 3:**
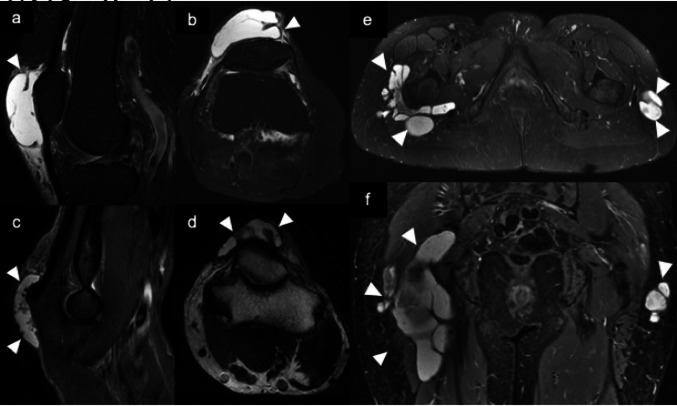
**a**, **b** Prepatellar bursitis. Axial (**a**) and sagittal (**b**) fat-suppressed T2-weighted MR images show a high signal intensity mass (arrow heads) located anterior to the patella. **c**, **d** Olecranon bursitis. Axial fat-suppressed T2-weighted (**c**) and sagittal T2-weighted MR images (**d**) demonstrate a flat-shaped cystic mass (arrowheads) located between the skin and underlying olecranon. **d**, **e** Trochanteric bursitis**.** Axial (**e**) and coronal (**f**) fat-suppressed T2- weighted images reveal a high signal intensity masses (arrowheads) surrounding the greater trochanter

The synovial membrane (synovium) is a connective tissue layer ranging from approximately 0.5 to 5 mm thickness. It lines the inner surface of diarthrodial joint capsules, tendon sheaths, and bursae. Synovial joints contain small amounts of synovial fluid enriched with hyaluronic acid [1]. In these joints, the articulating bone surfaces are covered with hyaline cartilage, with the exception of a small “bare area” located between the insertion of the joint capsule and the cartilage, where the bone is covered solely by synovium (Fig. 1) [2]. The synovial membrane lines movable (diarthrodial) joints, bursae, and tendon sheaths. Its primary role is not only acting as a specialized vascular tissue that lubricates and nourishes the articular structures but also serving as a shock absorber [3]. Tendon sheaths, also known as tenosynovium, enclose segments where tendons pass over joints or curve around bony prominences (Fig. 2) [4]. A detailed understanding of bursal anatomy is crucial for correctly identifying bursal pathologies. Subcutaneous bursae are located between the skin and underlying bony prominences, such as at the olecranon and patella. Subfascial bursae are situated between deep fascia and bone, while subtendinous bursae exist where one tendon passes over another (Fig. 3). Some bursae, when located adjacent to joints, may communicate directly with the joint cavity, forming a communicating bursa [5]. Such normal communication occurs, for instance, at the hip (iliopsoas bursa) and the knee (gastrocnemio-semimembranosus bursa). In contrast, pathological communication can develop at the glenohumeral joint (subacromial bursa) following a rotator cuff tear. Normal connections between the joint cavity and tendon sheaths may also be found, such as between the ankle joint and the flexor hallucis longus tendon sheath.

Many bursal lesions including tenosynovial giant cell tumor and synovial chondromatosis exhibit characteristic MRI findings. Awareness of these imaging features, combined with knowledge of their pathological and anatomical aspects, enables accurate diagnosis [5].

## Practical approach to differentiating intra-articular and extra-articular tumors

The distinction between intra-articular and extra-articular tumors relies on careful evaluation of anatomic origin and direct MRI findings. Intra-articular tumors are defined by their location within the joint space defined by the synovium, others arise from adjacent fat pad, bursae, cartilage, nerves, ligamentous or tendinous connections and are considered extra-articular [6, 7]. In general, intra-articular tumors do not spread outside the joint in the early stages but first spread within the joint. In cases where a lesion extends across both intra- and extra-articular compartments, the relative tumor volume in each compartment may serve as an important factor for determining the primary site of origin.

## Intra-articular tumor

### Tenosynovial giant cell tumor

Tenosynovial giant cell tumor (TSGCT) is the standardized term adopted in the 2020 World Health Organization (WHO) Classification of Soft Tissue and Bone Tumors (5th edition), though "giant cell tumor of tendon sheath" remains an acceptable alternative. This fibrohistiocytic neoplasm can present in either diffuse or localized forms, previously referred to as pigmented villonodular synovitis and giant cell tumor of tendon sheath, respectively.

**Fig. 4:**
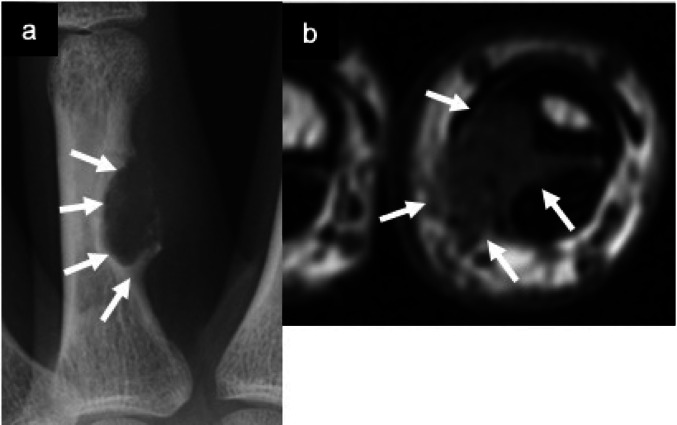
Localized type tenosynovial giant cell tumor. Plain radiograph of the finger (**a**) shows a soft tissue mass associated with pressure induced bony erosion and a surrounding sclerotic rim at the proximal phalanx (white arrows). Axial fat-suppressed T1-weighted image (**b**) demonstrates a soft tissue mass with corresponding bony erosion (white arrows)

**Fig. 5:**
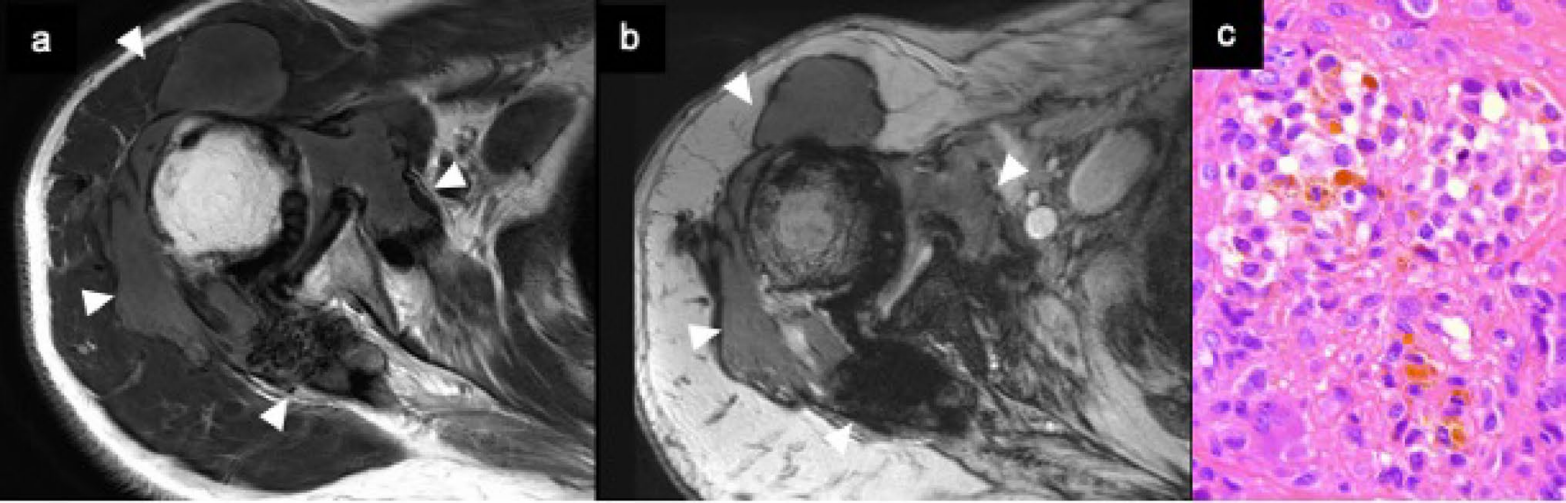
Diffuse type tenosynovial giant cell tumor. Axial T2-weighted MR image (**a**) shows diffusely thickened synovium surrounding the shoulder joint (arrowheads). Axial T2*-weighted MR image (**b**) demonstrates more pronounced low signal intensity, reflecting hemosiderin deposition. Photomicrograph (**c**) reveals proliferation of histocytes and abundant hemosiderin laden macrophages

TSGCT is characterized by a proliferation of villous and nodular synovial projections involving joints, bursae, or tendon sheaths. The localized type most commonly occurs in the hand, while the diffuse form frequently involves the knee, followed by the hip, ankle, and shoulder [8]. Localized TSGCTs typically arise as nodular, benign masses adjacent to the tendon sheath, whereas diffuse TSGCTs presents as infiltrative lesions involving the entire joint, leading to joint destruction and a high recurrence rate. [8]. Adjacent bone may exhibit compressive remodeling or reactive changes in the localized form (Fig. 4). Compressive bone resorption and reactive osteosclerosis are characteristic of adjacent slow-growing soft-tissue tumors. When tendon sheaths are affected, the lesion is termed "giant cell tumor of tendon sheath," whereas involvement of the entire joint synovium with prominent villous projections defines the diffuse type (Fig. 5). In diffuse TSGCT, bony erosions are frequently observed in joints with tight capsules, such as the hip, ankle, and elbow. Joint aspiration often reveals brown-colored fluid due to previous hemorrhage in diffuse cases. Both localized and diffuse types share a common molecular background of CSF1 rearrangement; however, their clinical courses differ markedly due to distinct sites of origin and growth patterns.

On computed tomography (CT), diffuse TSGCT appears as a synovial mass with slightly higher attenuation than that of surrounding muscle tissue, typically ranging from 55 to 75 Hounsfield Units (HU), reflecting hemosiderin accumulation [9].

MRI is the most crucial imaging modality for diagnosing TSGCT, assessing lesion extent, and guiding treatment planning [10]. The overall signal intensity of the diffuse TSGCT is similar to or less than that of skeletal muscle on T1-weighted MR images (T1WI). A similar pattern is seen on T2-weighted MR images (T2WI), although scattered areas of hyperintensity, representing fluid and congested synovium, may be present. Localized TSGCT tends to be low to iso signal intensity on T1WI, and iso to high signal intensity on T2WI [11]. It may occasionally exhibit foci of low signal on T1WI and T2WI due to the presence of hemosiderin within the mass [12]. Gradient-echo sequences (T2*-weighted imaging) frequently reveal blooming artifacts, manifesting as exaggerated hypointense areas due to hemosiderin deposits [10]. The decreased signal intensity is more pronounced at high field strength. On contrast-enhanced T1WI, the majority of signal intensities were heterogeneously enhanced [11]. Blooming artifact with marked hypointensity on T2*WI is a characteristic finding and serves as an important distinguishing feature from other diseases showing hypointense synovium on T2WI, such as amyloid arthropathy.

### Malignant tenosynovial giant cell tumor

Malignant tenosynovial giant cell tumor (MTSGCT) was first described by Castens et al. in 1979 [13]. The malignant counterpart of benign TSGCT is extremely rare [14]. Li et al., 2008 [15] reported the clinicopathologic characteristics of seven cases of malignant diffuse TSGCT, comparing them with 24 benign lesions and reviewing 23 previously published malignant cases. MTSGCT is defined either by the coexistence of overtly malignant areas within a benign TSGCT or by the recurrence of a typical TSGCT as a sarcoma [16]. Approximately half of patients with malignant diffuse-type TSGCT develop distant metastases and eventually succumb to the disease [15].

Most MTSGCTs involve the lower limbs, with a strong predilection for the knee. On CT, MTSGCT typically appears as a large, irregular soft tissue mass encasing the joint, with poorly defined margins. These tumors frequently exhibit extensive infiltrative bone destruction, reflecting their malignant nature [17–20].

**Fig. 6:**
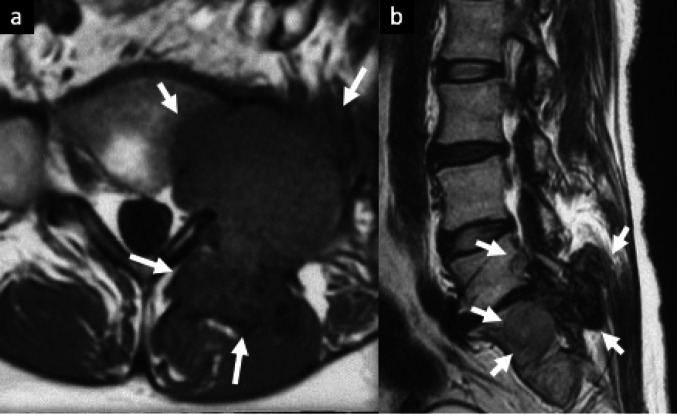
Malignant tenosynovial giant cell tumor arising in the facet joint**.** Axial T1-weighted MR image (**a**) demonstrates a low signal intensity mass with bony destruction involving the left L5/S1 facet joint (arrows). On T2-weighted sagittal MR image (**b**), low signal intensity in the posterior elements is more prominent (arrows)

MRI findings of MTSGCT are characteristically heterogeneous. The lesions typically exhibit a predominantly muscle-like signal intensity on T1WI. Areas of low signal intensity on both T1WI and T2WI may reflect iron-containing hemosiderin deposits associated with necrosis or cystic changes within the tumor. [12, 18, 19]. Although imaging of MTSGCT has only rarely been reported, extensive bone marrow invasion should be viewed with suspicion for malignancy (Fig. 6).

### Synovial chondromatosis

**Fig. 7:**
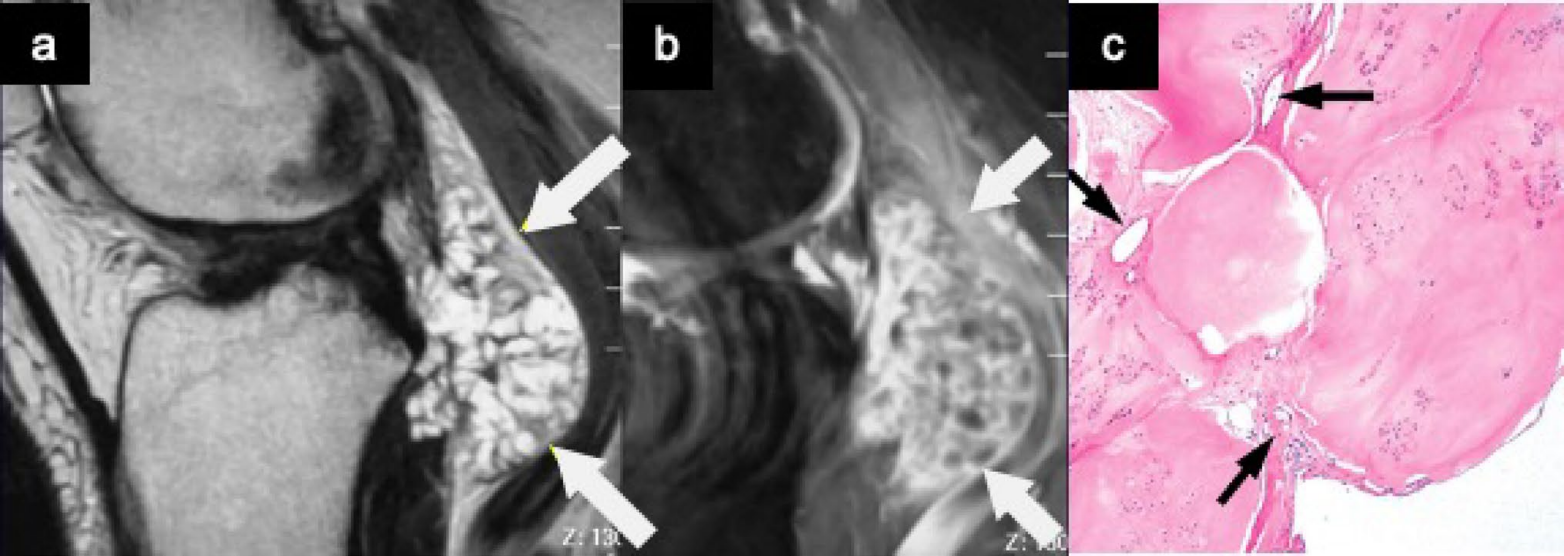
Synovial chondromatosis. Sagittal T2-weighted MR image (**a**) shows a hyperintense mass with hypointense septa in posterior aspect of intercondylar notch (arrows). Sagittal fat-suppressed postcontrast T1-weighted MR image (**b**) demonstrates the peripheral and septal enhancement of the mass. Photomicrograph (**c**) reveals hyaline cartilage in a lobular arrangement separated by fibrous septa with capillary vessels (arrows)

**Fig. 8:**
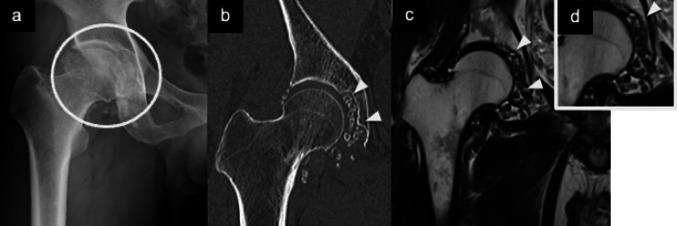
Synovial chondromatosis. Plain radiograph (**a**) and coronal CT (**b**) show multiple round and speckled calcifications (arrowheads) surrounding the proximal aspect of the right femur. Coronal T2-weighted MR images (**c**, **d**) demonstrate multiple low-signal foci (arrowheads), some with central high signal intensity, suggestive of fatty marrow within calcified nodules

Synovial chondromatosis is a locally aggressive neoplastic process characterized by the formation of multiple hyaline cartilaginous nodules within the joint space, subsynovial tissue, or, in extra-articular cases, within the tenosynovium [21]. The knee is most frequently affected, followed by the hip, shoulder, and elbow [22]. Some cases are confined entirely to extra-articular locations, commonly referred to as tenosynovial chondromatosis, which typically arises in the hands and feet [23]. These lesions must be distinguished from joint effusion or soft tissue tumors. MRI findings vary depending on the disease stage and the degree of calcification within the nodules. In early stages without calcification, nodules appear isointense to muscle on T1WI and hyperintense on T2WI. They often manifest as intra-articular conglomerate masses. The presence of internal septations and peripheral or septal enhancement following intravenous gadolinium administration can aid in establishing the diagnosis (Fig. 7). When calcification is present, the radiographic features of synovial chondromatosis become pathognomonic, typically revealing multiple intra-articular calcified nodules of relatively uniform size. In more advanced cases, mature bone formation may occur within a nodule, sometimes containing fatty marrow. On T1WI and T2WI, this presents as a ring-like structure with a central area of high signal intensity (Fig. 8) [6].

### Synovial chondrosarcoma

**Fig. 9:**
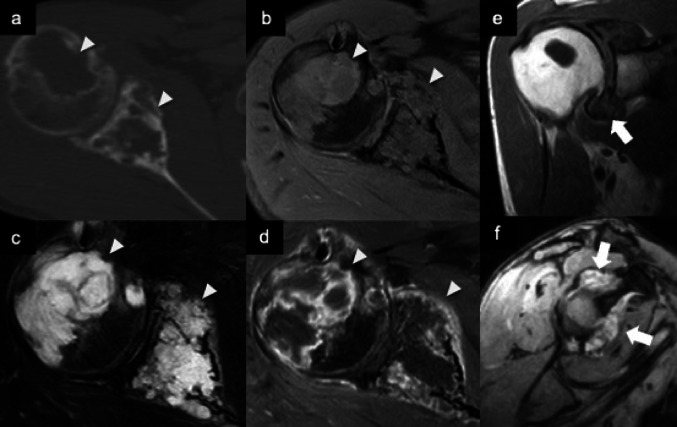
Synovial chondrosarcoma. Axial CT bone image (**a**) shows osteolytic lesion (arrowheads) of the humeral head and scapula. Axial fat-suppressed T1-weighted MR image (**b**) demonstrates lobulated intramedullary lesions in both scapula and humerus (arrowheads). Axial fat-suppressed T2-weighted MR image **c** reveals high signal intensity of the lesion, and fat-suppressed postcontrast T1-weighted MR image (**d**) shows peripheral gadolinium enhancement suggesting chondroid nature (arrowheads). Sagittal T1-weighted MR image (**e**) and oblique sagittal T2*-weighted MR image (**f**) obtained 6 years earlier revealed a diffuse synovial lesion of the intra-articular space (arrows)

Synovial chondrosarcoma is a very rare entity and is found in most of cases in association with synovial chondromatosis. For this reason, it is believed that synovial chondrosarcoma usually is a malignant transformation of a synovial chondromatosis [24]. Although rare, malignant transformation should be considered in cases of rapid recurrence following synovectomy. [25]. Radiographs, CT, and MRI may demonstrate a permeative and destructive bone pattern rather than the typical erosive changes seen in synovial chondromatosis [26]. Synovial chondrosarcoma and primary synovial chondromatosis may have radiologically similar appearances, including extrinsic bony erosion. However, true cortical destruction with bone marrow invasion and permeation is a feature that should be considered a sign of malignancy (Fig. 9) [27].

### Synovial hemangioma / Intraarticular venous malformation

Synovial hemangioma is a benign vascular proliferation that arises from a synovium-lined surface, almost exclusively affecting the knee joint, with the suprapatellar bursa being the most common site [28]. The terminology of these lesions is still ill-defined; although they have been widely described as synovial hemangiomas in the literature, most recent reports refer to them as intraarticular venous malformations (IAVMs) [29]. Repeat episodes of intra-articular hemorrhage due to synovial hemangioma may lead to synovial hyperplasia and hemosiderin deposition, hence careful assessment of imaging findings is important as not to confuse with diffuse TSGCT.

**Fig. 10:**
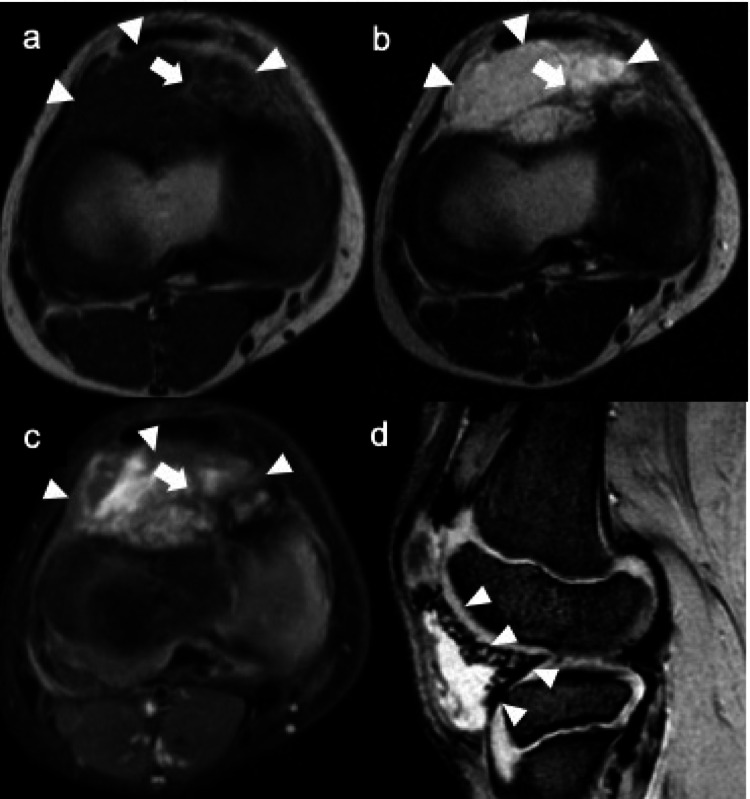
Synovial hemangioma. Axial T1-weighted MR image (**a**) shows heterogenous intensity mass (arrowheads) containing high signal areas (arrow) in Hoffa’s fat pad. On axial T2-weighted MR image (**b**), the mass is high signal intense to subcutaneous fat. Fat-suppressed postcontrast MR image (**c**) shows heterogenous enhancement. Sagittal T2-weighted MR image (**d**) shows a hyperintense mass with low signal intensity septa in Hoffa’s fat pad (arrowheads) and thick synovium with low signal intensity, representing hemosiderin deposits due to repeated intra-articular hemorrhage

MRI findings of synovial hemangioma are often pathognomonic, typically presenting as a lobulated intra-articular mass with characteristic signal features. The lesion usually demonstrates intermediate signal intensity on T1WI and markedly high signal intensity on T2WI, which likely reflects blood pooling within vascular spaces. Low signal intensity linear structures seen within the mass on T2WI are thought to represent fibrous septa or vascular channels [6, 30, 31] (Fig. 10). Fibro-fatty septa can often be recognized as showing high signal intensity on T1WI and intermediate intensity on T2WI. In cases of intra-articular hemorrhage, the synovium may become thickened with prominent hemosiderin deposition, which can make differentiation from TSGCT challenging. Therefore, careful evaluation for the presence of the vascular lesion within or adjacent to the joint is essential.

When evaluating thickened synovium with hemosiderin deposition, hemophilic arthropathy should be included in the differential diagnosis. Hemophilic arthropathy typically shows diffuse synovial hypertrophy without a discrete intra-articular mass showing hyperintensity on T2WI, which aids in differentiation [32].

### Lipoma arborescens

Lipoma arborescens is a rare intra-articular lesion characterized by the replacement of subsynovial tissue with mature adipocytes, resulting in villous synovial proliferation [33]. It typically presents as a monoarticular condition, most frequently affecting the knee joint, particularly the suprapatellar pouch [34]. Patients are usually in their fifth to seventh decades of life and often present with a long-standing, painless, and slowly progressive swelling of the joint, accompanied by joint effusion [33]. This lesion is frequently associated with degenerative joint disease, joint trauma, and diabetes mellitus.

**Fig. 11:**
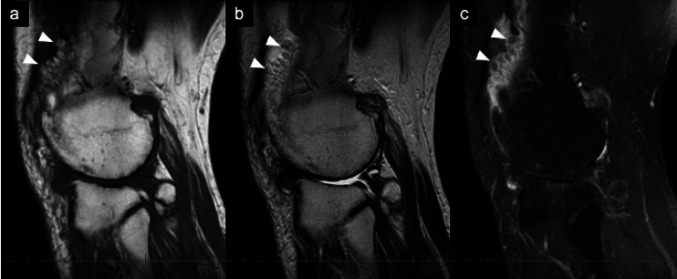
Lipoma arborescens**.** Sagittal T1-weighted (**a**) and T2-weighted (**b**) MR images show a frond-like mass within a suprapatellar bursa that has a signal intensity comparable to that of fat (arrows). Degenerative change of the knee joint is also seen. Sagittal fat-suppressed postcontrast T1-weighted image (**c**) shows marked gadolinium enhancement of the synovium excluding the fat mass (arrowheads)

The characteristic MRI findings of lipoma arborescens include: (i) joint effusion; (ii) a synovial mass with a frond-like or arborescent appearance showing signal intensity equivalent to that of fat on all sequences, with signal suppression on fat-suppressed images; (iii) absence of hemosiderin deposition; and (iv) no post-contrast enhancement of the mass itself, although enhancement of the synovium may be observed, suggestive of chronic synovitis (Fig. 11) [6, 35–37]. These MR findings can allow specific preoperative diagnosis. Although the etiology of lipoma arborescens has not been clearly defined, it has been considered more likely to be a reactive process of the synovial tissue, which could be associated with joint trauma, meniscal lesions, chronic synovitis or arthritis, rather than a neoplastic lesion [38, 39].

### Synovial sarcoma

Synovial sarcoma is the third most frequent soft-tissue sarcoma in adults, accounting for roughly 10% of all soft-tissue sarcomas [40]. Approximately 70% arise in the deep soft tissues of the extremities, most commonly in juxta-articular locations of adolescents and young adults, whereas about 15% occur in the trunk and 7% in the head and neck region [41]. Primary intra-articular synovial sarcoma is extremely rare, reported in approximately 3–6% of synovial sarcomas in large case series [42, 43]. Despite its name, synovial sarcoma does not originate from synovial tissue but arises from undifferentiated mesenchymal cells [6]. On CT, synovial sarcomas typically appear as heterogeneous, deep-seated soft-tissue masses with attenuation similar to or slightly lower than that of skeletal muscle. Calcifications are observed in nearly one-third of cases in large series [44]. MRI usually reveals a mass exceeding 5 cm located near a joint, situated deep in the soft tissues. A characteristic “triple signal” on T2-weighted images, comprising solid cellular elements (intermediate signal intensity), hemorrhagic or necrotic areas (high signal intensity), and calcified or fibrotic collagenized regions (low signal intensity), may also be identified [44]. Large lesions tend to be more noticeable the signal pattern. Although the radiographic features are not pathognomonic, findings of a soft-tissue mass if calcified and near but not in a joint of a young patient, are suggestive of the diagnosis.

### Other sarcoma and metastases arising from nonspecific sites

**Fig. 12:**
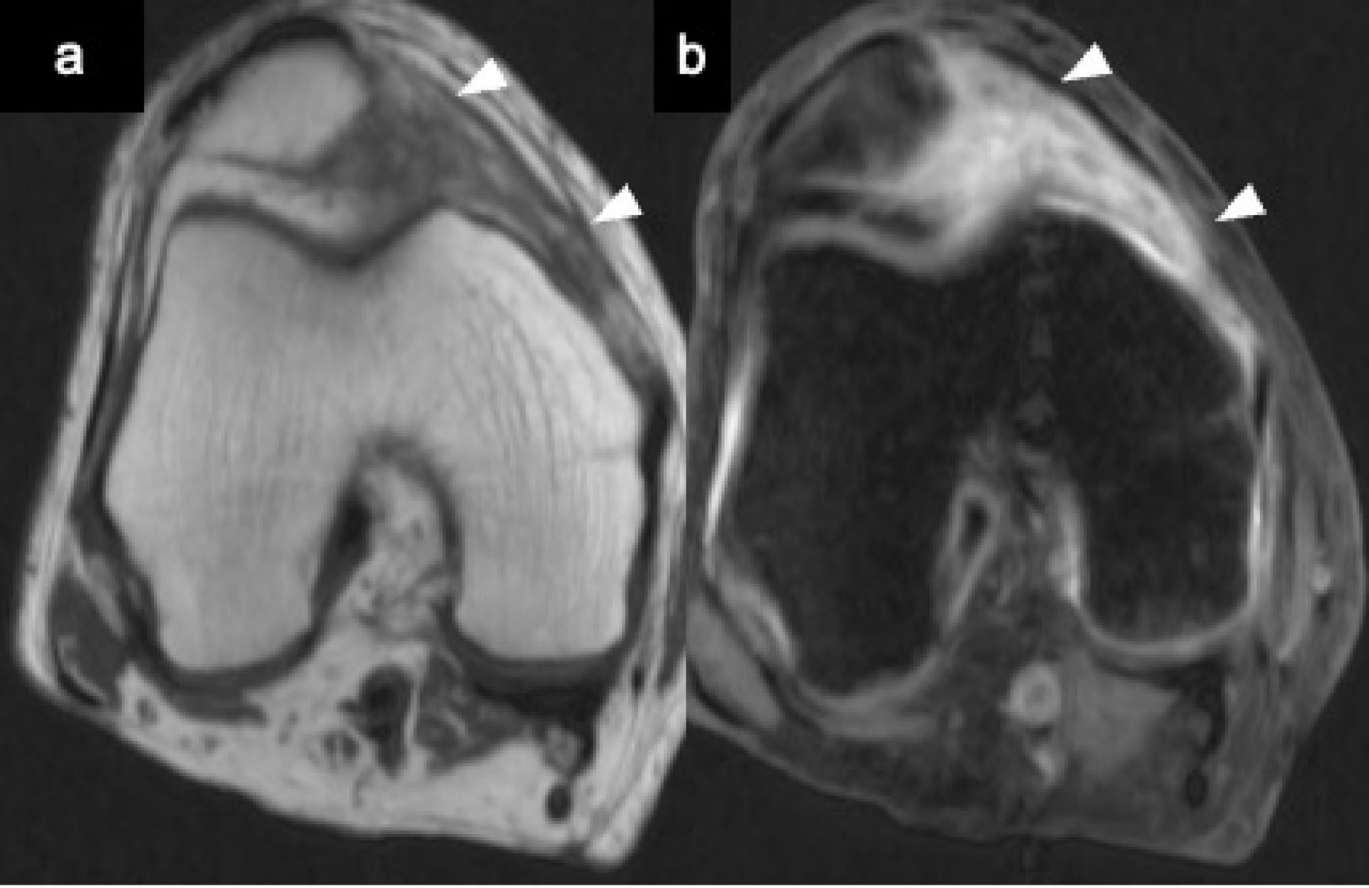
Metastasis from salivary gland cancer. Axial T1-weighted MR image (**a**) shows an ill-defined soft tissue mass (arrowheads) anterior to the lateral condyle of the femur. Marked enhancement is seen on fat-suppressed T1-weighted MR image obtained after contrast administration (**b**)

Primary intra-articular sarcomas are exceedingly rare and usually present with non-specific symptoms such as pain or swelling (Fig. 12). Synovial metastasis is also rarely occurred. The condition usually has a poor prognosis, with average patient survival of less than 5 months. Adenocarcinoma is the most common histological type and the knee is the most frequently affected joint [6, 45]. Although rare, primary intra-articular sarcomas/metastases should be considered when an intraarticular solid mass is identified that does not exhibit the clinical or imaging characteristics of representative intra-articular lesions as described above.

In Table 1, we have provided a comprehensive summary of intra-articular tumor / tumor-like lesions, including their epidemiology, imaging characteristics, and preferred diagnostic modalities.

**Table 1:** Summary of the key characteristics of intra-articular tumor/tumor—like lesions

Lesion / Disease	Age (Peak)	Common Sites	Characteristic Imaging Findings	Useful Modalities / Sequences	Notes / Key Points
Localized TSGCT	30–50y	Hand	T2/GRE blooming (hemosiderin), lobulated synovial mass	Occasionally MRI (GRE, T2*)	Local recurrence, Pressure erosion
Diffuse TSGCT	< 40y	Knee, Hip		Generally MRI (GRE, T2*)	Joint destruction, High recurrence
Malignant TSGCT	50-60y	Lower limbs, Knee		MRI (GRE, T2*) + CT for bony change	Extensive bone marrow invasion
Synovial Chondromatosis	30–50y	Knee, Hip	With calcification (Multiple cartilaginous nodules) Without calcification (internal septations and peripheral or septal enhancement	With calcification MRI, CT and radiography Without calcification MRI including postcontrast image	Multiple intra-articular calcified nodules of relatively uniform size
Synocvial Chondrosarcoma	< 55y	Knee, Hip	Extrinsic bony erosion	MRI + CT for bony change	Rapid recurrence following synovectomy
Synovial Hemangioma / IAVM	Children– adolescents	Knee (suprapatellar bursa)	T1 intermediate, T2 high	MRI T2WI or STIR for the lesion	Fibro-fatty septa
Lipoma Arborescens	50–70y	Knee (suprapatellar pouch)	Frond-like fat signal lesion	MRI (FS T1/T2)	Often associated with degenerative joint disease,
Synovial Sarcoma	adolescents or young adults	Deep soft tissue of the lower and upper extremities	Heterogeneous mass, triple signal on T2WI, calcifications (30%)	MRI CT and radiography for detecting calcification	“Triple signal” on T2WI Generally near but not in a joint
Other Sarcomas / Metastasis	Varies	Any joint /periarticular	Aggressive soft tissue mass, bone destruction, heterogeneous enhancement	MRI + Biopsy Surveillance for primary lesion (PET, etc.)	Rule out metastasis if multiple lesions

WI, weighted image, CT, computed tomography, MRI, magnetic resonance imaging, TSGCT, tenosynovial giant cell tumor, IAVM, intra-articular venous malformation, FS, fat suppressed, GR, gradient echo

## Other synovial disease

In addition to the representative intra-articular tumors summarized above, various non-neoplastic and other neoplastic diseases can also form mass-like lesions within the joint. Herein, we describe the imaging findings of deposition diseases (such as gout and amyloid arthropathy), autoimmune conditions (such as rheumatoid arthritis), miscellaneous lesions (including ganglion cysts and cyclops lesions), as well as sarcomas and metastases originating from nonspecific sites.

### Gout

Gout is a metabolic disorder characterized by hyperuricemia, acute episodes of inflammatory arthritis, and the deposition of monosodium urate monohydrate crystals (referred to as tophi) in and around joints. Tophaceous gout involves localized crystal aggregates embedded within a proteinaceous matrix and surrounded by intense inflammation. The chronic form typically affects men in their fifth to seventh decades, with the predilection for the small joints of the hands and feet [46]. Diagnosis during the acute stage is usually determined through clinical and laboratory assessment. However, in the chronic form, the diagnosis may be difficult but can be assisted by the use of CT or MRI.

**Fig. 13:**
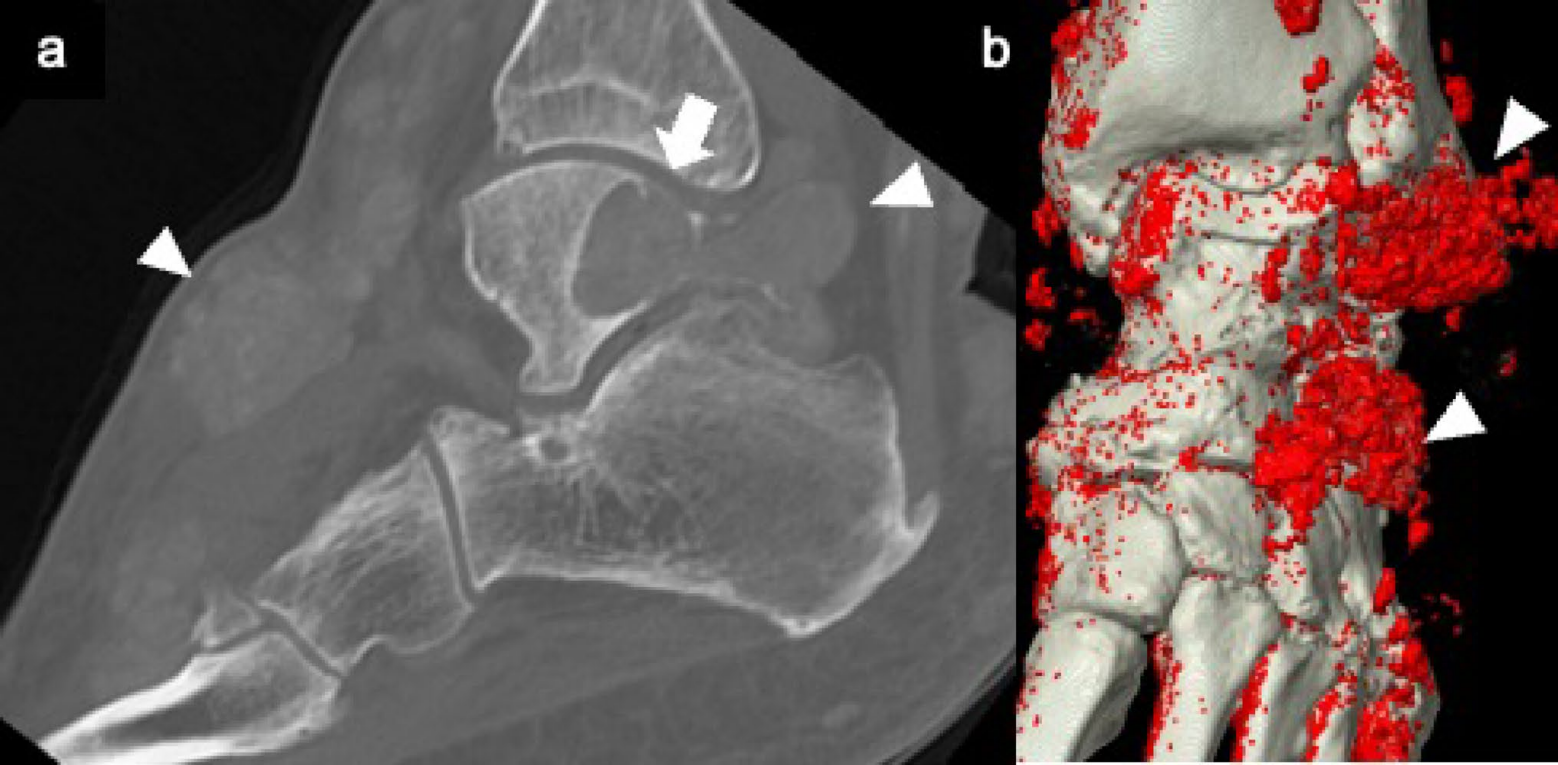
Gout. Sagittal CT bone image (**a**) shows dense mineralized soft tissue masses (arrowheads) and characteristic juxta-articular erosions with overhanging edges (arrows), typical of chronic gout. A color-encoded material decomposition image obtained with dual energy CT (**b**) highlights multiple urate crystal depositions surrounding the ankle joint (arrowheads)

Radiographically, gout is characterized by well-defined erosions with overhanging edges, preservation of joint space, absence of periarticular osteopenia, and the presence of soft tissue nodules. On MRI, the lesions typically demonstrate homogeneous low signal intensity relative to muscle on T1WI and low to intermediate heterogeneous signal intensity on T2WI [47, 48]. Dual-energy CT (DECT), which exploits the differential attenuation properties of urate crystals versus calcium, can be particularly useful in cases of atypical clinical presentation, discordant serum urate levels, or when distinguishing acute flares from chronic changes (Fig. 13) [49]. The tophi typically appear as distinct soft tissue masses with a attenuation of 160-170HU, exceeding that of the surrounding soft tissues [50]. The presence of high attenuation on CT provides a valuable diagnostic clue for differentiating it from other lesions.

### Amyloid arthropathy

Amyloid arthropathy is particularly common in patients undergoing long-term hemodialysis, with risk increasing as longer dialysis vintage [51]. Articular involvement is usually bilateral and commonly affects the shoulders, hips, knees, and wrists [52].

**Fig. 14:**
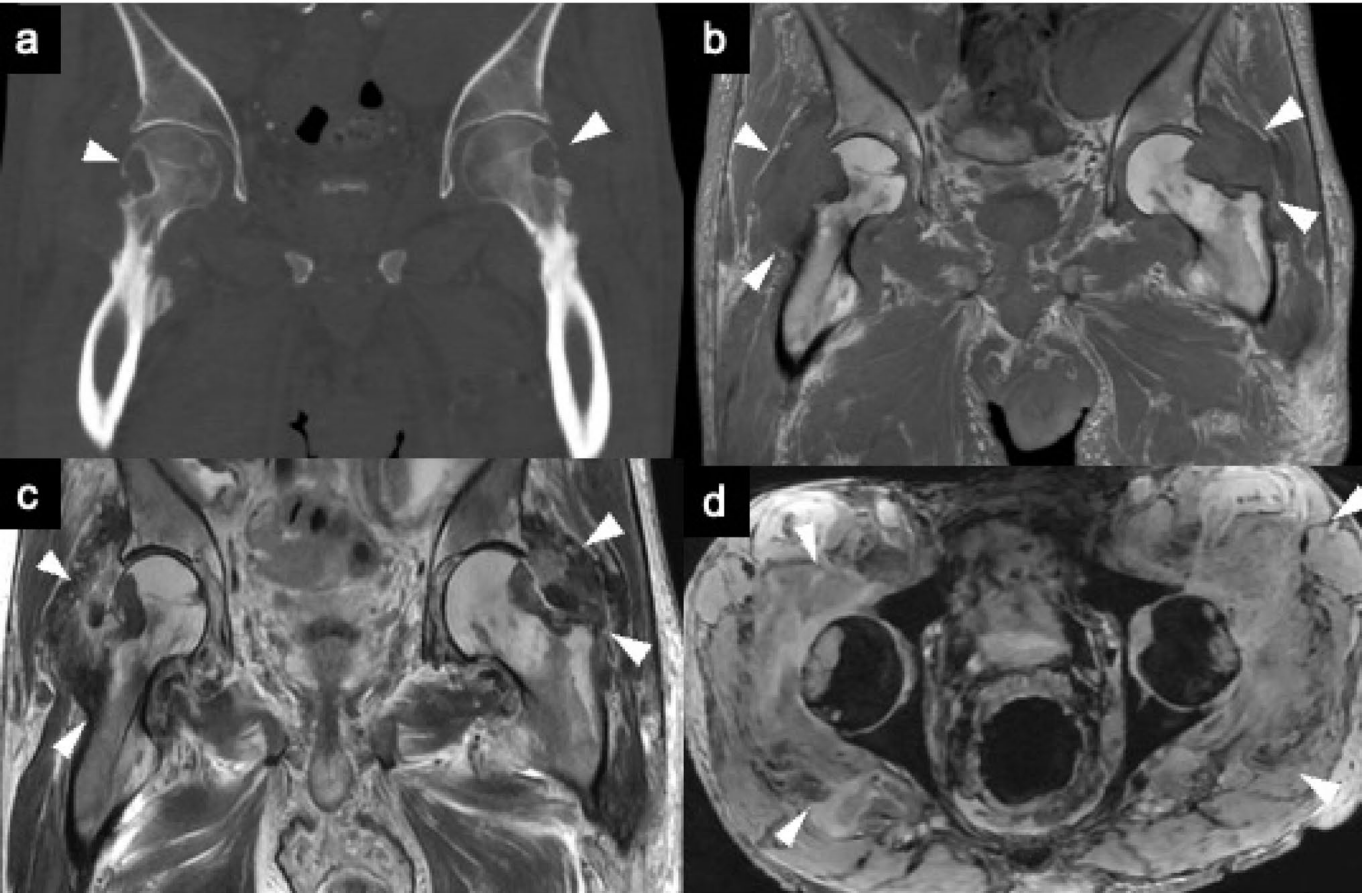
Amyloid arthropathy. Coronal T1-weighted MR image (**a**) shows diffuse soft tissue swelling around both hips, extending into subchondral bones (arrowheads). Coronal CT (**b**) reveals bone erosions with sclerotic margins (arrowheads). Axial T2-weighted (**c**) and T2*-weighted (**d**) images demonstrate intraarticular masses (arrowheads), which do not exhibit a paramagnetic effect, as evidenced by the absence of signal drop on the T2*- weighted image

Amyloid deposits in a joint synovium are seen on CT and MRI as soft tissue masses. Extrinsic erosion of bone is not uncommon, particularly in the hip and shoulder joints. MRI typically shows abnormal soft-tissue deposition with low to intermediate signal intensity on both T1WI and T2WI [52], reflecting the collagen-like properties of amyloid. These lesions do not demonstrate a paramagnetic effect on gradient-echo sequences, which can aid in differentiating amyloid arthropathy from hemosiderin-rich synovial disorders such as diffuse-type TSGCT (Fig. 14) [6, 53].

### Rheumatoid arthritis

Rheumatoid arthritis is a systemic inflammatory disorder of unknown etiology that primarily affects synovial tissue. It is hypothesized that cross-reactive antibodies against an unidentified antigen are deposited in the synovium, leading to a proliferative and hypervascular tissue known as pannus. Although the pannus is largely composed of fibrous tissue, it may have signal intensity similar to that of joint fluid on unenhanced MRI. The disease typically presents as symmetrical polyarthritis affecting the synovial joints of the appendicular skeleton, with prominent involvement of the proximal interphalangeal and metacarpophalangeal joints of the hands, the wrists, the metatarsophalangeal joints of the feet, the knees, and the elbows, and the glenohumeral and acromioclavicular joints.

**Fig. 15:**
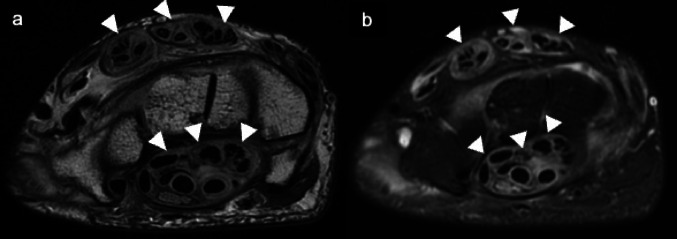
Rheumatoid arthritis. Axial T2-weighted (**a**) and fat-suppressed T2-weighted MR images (**b**) show thickened tendon sheaths surrounding the flexor and the extensor tendons (arrowheads)

Postcontrast MRI enable the differentiation of fibrous and hypervascular pannus, as well as the identification of large joint effusions. [54] (Fig. 15). It is recommended that imaging be performed shortly after contrast medium injection so as to avoid diffusion of the contrast material from the synovium into the joint fluid. MRI reveals pannus with intermediate to low signal intensity on T1WI and T2WI [53]. Associated features such as soft tissue swelling, periarticular osteopenia, marginal erosions, and diffuse joint space narrowing further support the diagnosis [55]. In addition, the distribution of synovial joint involvement contributes to an accurate diagnosis. RA and tuberculous arthritis may have similar clinical characteristics, consisting of a chronic course with periarticular soft-tissue swelling, as well as similar radiologic findings, such as periarticular osteoporosis, bone erosion, and presence of joint effusion, rendering differential diagnosis difficult [56]. Uneven and thick synovial proliferation is more commonly observed in RA, whereas even and thin synovial thickening, larger bone erosions with peripheral rim enhancement, and the presence of extra-articular cystic masses indicating abscess are more frequently seen in tuberculous arthritis, which typically presents as a monoarticular process. MRI evaluation can therefore be useful in differentiating between these two entities [57].

### Ganglion

Intra-articular ganglion of the knee is seen in 0.2–1.9% of MRI examinations [58–60], with a male-to-female ratio of approximately 3:1[61]. They typically arise from the cruciate ligaments, although they may also originate from the infrapatellar fat pad [60, 61].

**Fig. 16:**
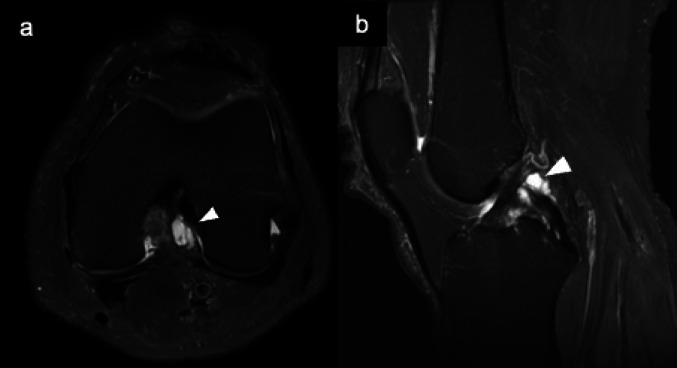
Intra-articular ganglion. Axial (**a**) and sagittal (**b**) fat-suppressed T2-weighted MR images demonstrate a septated, high signal intensity mass (arrows) located on either side of the posterior cruciate ligament (PCL)

 MRI typically shows well-circumscribed lesions with fluid-equivalent signal intensity. Posterior cruciate ligament (PCL) ganglion is often multilocular and well defined. In contrast, anterior cruciate ligament (ACL) ganglion tends to have a fusiform appearance, aligned along the ligament fibers (Fig. 16) [58, 59, 61, 62]. Ganglion and synovial cysts have similar MRI appearances but differ in their pathological characteristics. Synovial cysts possess a synovial lining and often communicate with the joint cavity, while ganglion cysts lack a true lining and arise from myxoid degeneration of connective tissue. The presence of joint communication and association with arthropathy favor a synovial cyst, whereas a wrist or tendon-sheath origin suggests a ganglion [63].

### Postoperative intraarticular tumor

After orthopedic interventions—particularly arthroscopic or reconstructive procedures—various mass lesions may develop within or around the joint. These lesions are not true neoplasms but represent reactive or reparative processes, including localized arthrofibrosis (cyclops lesion), postoperative hematomas, and foreign-body granulomas. Their clinical importance lies in mimicking recurrent or residual tumors, producing mechanical symptoms such as limited range of motion, and sometimes causing diagnostic confusion on imaging studies [64, 65].

**Fig. 17:**
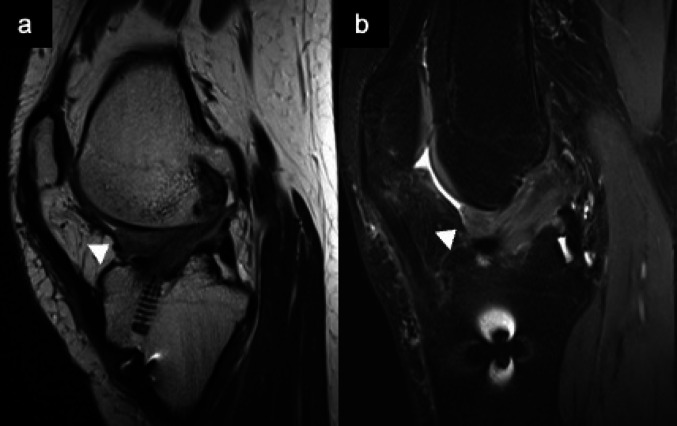
Cyclops lesion (Teenage female operated the ACL before 10 months). Sagittal T2-weighted (**a**) and fat suppressed T2-weighted (**b**) MR images demonstrate an intermediate signal intensity mass (arrowheads) located anterior to the intercondylar notch, near the tibial insertion of the cruciate ligaments

Localized anterior arthrofibrosis, commonly referred to as a cyclops lesion, occurs following ACL reconstruction in less than 10% of cases [6, 66]. On arthroscopy, these fibrous nodules have a bulbous morphology with reddish-blue discoloration, resembling an eye, hence the term “cyclops.” Histologically, the lesion consists of a central core of granulation tissue surrounded by dense fibrous tissue [67]. MRI demonstrates a low to intermediate signal intensity nodular lesion on T1WI, located anterior to the intercondylar notch near the tibial insertion of the graft. On T2WI, the lesion appears heterogeneous but predominantly of low to intermediate signal intensity, consistent with its fibrous content (Fig. 17) [66, 67].

Hematomas demonstrate variable MRI signal depending on the age of the blood products, although most of them contain areas of hyperintensity on T1WI. Contrast administration is therefore helpful in showing the characteristic peripheral rim enhancement of the hematoma. A foreign-body granuloma typically appears as a soft-tissue mass with contrast enhancement surrounding the retained foreign body. On MRI, it usually demonstrates low signal intensity on both T1- and T2WIs [68].

## Conclusion

Many synovial proliferative disorders demonstrate distinctive MRI characteristics. Awareness of these imaging findings, along with knowledge of their underlying pathological and anatomical correlates, facilitates accurate diagnosis. Even though a broad spectrum of diseases may affect the synovium, thorough MRI evaluation in conjunction with clinical information allows for a focused and effective differential diagnosis.
